# St. Bartholomew's Hospital

**Published:** 1830-05

**Authors:** 


### HOSPITAL REPORTS.

### ST. BARTHOLOMEW'S HOSPITAL.

### *Tapping in Hydrocephalus.*

Dr. Conquest introduced to his class, at St. Bartholomew's Hospital, on Saturday evening, one of the two children who have been successfully tapped by him for the relief of water in the head. It having been previously intimated that the child would be brought forward, considerable interest was excited, and an unusual number of gentlemen were present. This child, a girl of about two years of age, had several signs of hydrocephalus from a date soon after its birth, and for many months past the head had gradually increased, until it acquired an enormous size. The forehead was singularly broad, and the anterior fontanelle unnaturally large. The pupils were permanently dilated; the child slept almost incessantly, and frequently had two or three frightful convulsions during the day and night. Dr. Conquest operated some time since, before a large number of the pupils of the hospital, by pushing a very beautifully constructed trocar into the right lateral ventricle. He introduced it obliquely, close to the edge of the right temporal bone, about midway between the crista galli process of the ethmoid bone and the anterior fontanelle, so as to avoid the longitudinal sinus on the one hand, and the corpus striatum on the other. The instrument entered about two inches below the scalp. An ounce and a half of bloody serum, mixed with portions of cerebrum, escaped. The pulse became feeble, and temporary collapse followed. The fluid was allowed to escape stillicidium; and, within eight and forty hours, about two pints and a half flowed out of the opening. Almost immediately after the operation, the pupils became sensible to the stimulus of light; the drowsiness was succeeded by disinclination to sleep; and the pulse, which had always before been remarkably slow, became about eighty-five. Two days after the operation, the brain evidenced signs of inflammation, with high constitutional disturbance; and great alarm was excited by a rather formidable attack of convulsions. Leeches to the temples, and the constant application of cold to the head, subdued the local inflammation, and within four and twenty hours all became tranquil. The head was well strapped, and from the cessation of cerebral excitement no unfavorable circumstance occurred.

When this interesting child was exhibited to the class, every one was struck with the improvement of its appearance, and by the intelligence and cheerfulness of its countenance. Dr. C. stated that he considered the child perfectly well, and as exhibiting a most gratifying and triumphant proof that this seemingly formidable proceeding might be safely and successfully adopted under similar circumstances.

The other case, to which the Doctor has often adverted during the winter, he operated on last autumn, assisted by Dr. Hodgkin, the talented pathologist of Guy's Hospital. Nine ounces of serum were withdrawn from the posterior fontanelle. The head became lessened six inches in its circumference, and no increase in its size has yet recurred.

